# Carbohydrate accumulation patterns in mangrove and halophytic plant species under seasonal variation

**DOI:** 10.1038/s41598-024-72627-1

**Published:** 2024-09-14

**Authors:** Dhruvisha Mehta, Sandip Gamit, Dushyant Dudhagara, Vijay Parmar, Ashish Patel, Suhas Vyas

**Affiliations:** 1 Department of Life Sciences, Bhakta Kavi Narsinh Mehta, University, Khadiya, Junagadh, Gujarat 362263 India; 2 Department of Life Sciences, Hemchandracharya North Gujarat University, Patan, India

**Keywords:** *Sueda Nudiflora*, *Aeluropus lagopoides*, *Avicennia marina*, Seasonal variation, Abiotic stress resilience, Environmental adaptation, Physiology, Plant sciences

## Abstract

This study investigates the impact of carbohydrate accumulation in mangrove and halophytic plants on their response to abiotic stress. Using soil analysis and FT-IR spectroscopy, key species (*Sueda nudiflora*,* Aeluropus lagopoides*,* Avicennia marina*) were examined for seasonal changes in sugar content (reducing sugars, total soluble sugars, starch). The elevated carbohydrate levels may serve as an indication of the plant’s ability to adapt to different environmental conditions throughout the year. This accumulation enables plants to adapt to variations in their environment, assuring their survival and functionality during periods of environmental fluctuation. Halophytic plants’ sugar content peaked during the monsoon, suggesting biotic adaptations. The mangrove *Avicennia marina* had year-round sugar levels. PCA and Hierarchical Cluster Analysis revealed sugar accumulation trends across species and seasons. Partial Least Squares (PLS) analysis revealed correlations between soil characteristics and sugar content, suggesting plant-microbe interactions. K-means clustering and correlation analysis of FT-IR data revealed sugar composition and resource allocation trade-offs. These findings shed light on the role of carbohydrate metabolism in enabling coastal plants to endure stress. Gaining insight into these mechanisms can enhance sustainable agriculture in challenging environments and shed light on plant adaptations to evolving environmental conditions, especially biotic interactions.

**Supplementary Information:**

The online version contains supplementary material available at 10.1038/s41598-024-72627-1.

## Introduction

Halophytes and mangroves are fundamental elements of coastal ecosystems, playing a significant role in advancing environmental sustainability^1^. Adapted to saline tidal habitats, mangroves represent sturdy coastal flora that depend on effective sugar accumulation mechanisms for their survival^2^. Conversely, halophytes, thriving in high salinity settings^3^, have evolved specialized mechanisms to regulate their osmotic potential by accumulating sugars in order to sustain water uptake and turgor pressure in response to seasonal variations like increased or decreased salinity or drought. This adaptation enables plants to endure and acclimatise to unfavourable environmental conditions^4,5^. These botanical species support ecosystem stability, coastal defense, carbon storage, and biodiversity improvement^1,6^. Their resistance to unfavorable environmental conditions places halophytes and mangroves as crucial participants in both climate change mitigation and adaptation strategies^7^.

*Avicennia marina*, *Suaeda maritima*, and *Aeluropus lagopoides* have developed specific adaptations to their saline environments that are indicative of their evolutionary background. *Avicennia marina* has developed specific adaptations, such as salt filtration and pneumatophores, to successfully survive in the demanding intertidal areas of mangrove forests. These adaptations are essential for sediment stabilization and coastal protection. *Suaeda maritima* has developed physiological and biochemical adaptations to cope with high salinity levels in salt marshes. These adjustments enable the plant to effectively handle osmotic stress, thereby enhancing the resilience of salt marsh ecosystems. *Aeluropus lagopoides*, which has evolved in saline deserts and coastal dunes, have developed mechanisms to tolerate drought and salt. These mechanisms allow it to survive in extreme conditions and help to stabilize arid and saline landscapes. These evolutionary adaptations emphasize the species’ functions in preserving ecological equilibrium in their respective habitats.

These species were studied based on their critical roles in coastal ecosystems and their unique ability to adapt to saline environments. *Sueda nudiflora* exhibits remarkable salinity tolerance and possesses the capacity to accumulate carbohydrates as osmolytes, rendering it a suitable candidate for investigating stress responses. *Aeluropus lagopoides*, a distinct type of salt-tolerant plant, provides valuable information on the mechanisms by which non-succulent halophytes cope with osmotic stress. *Avicennia marina*, a representative species of mangrove, offers a contrasting example due to its distinctive adaptations to waterlogged and saline environments. This excerpt provides a thorough comprehension of carbohydrate accumulation in various plant species and their reactions to environmental stressors. Plant architecture and size significantly impact carbohydrate accumulation across different organs and species of mangrove and halophytes. Plants with diverse forms, such as leaf sizes, root structures, and stem types, have different strategies for carbohydrate storage and allocation. Larger plants with larger organs have greater storage capacities, while smaller plants with compact growth forms allocate carbohydrates more efficiently. Organs like leaves, stems, and roots have specialized roles in carbohydrate metabolism, affecting their adaptability to environmental conditions and resource availability.

The primary purpose of the experiment was to investigate and evaluate the patterns of carbohydrate accumulation among three different plant species—*Sueda nudiflora*, *Aeluropus lagopoides*, and *Avicennia marina*—during these three distinct seasons: winter, summer, and monsoon. The seasonal variability in carbohydrate metabolism was investigated over the course of two years, and multiple seasonal cycles were investigated in order to capture this variability. This approach not only makes it possible to gain an understanding of the adaptive strategies that these plants employ, but it also highlights the ecological roles that these plants play in coastal habitats that are subject to a variety of climatic influences. A crucial aspect of understanding the survival and adaptation strategies of halophytes and mangroves in challenging environments involves studying sugar accumulation and distribution patterns in various above-ground plant parts of saline plants, including pneumatophores, stems, and leaves from the Ghogha coastline of Bhavnagar^8^. The main aim of this study is to explore the responsible mechanisms for sugar accumulation in these plants to elucidate the seasonal variations in sugar levels that are crucial for stress tolerance, and energy production, serving as vital components for osmoregulation^2,9,10^.

The leaves of saline plants, pivotal in sugar production through photosynthesis, facilitate the synthesis of glucose and other sugars^11^. Stems, acting as sugar storage organs, contribute to sugar accumulation. The presence of pneumatophores in mangroves supports sucrose accumulation and aids in oxygen uptake, enhancing survival. Glucose serves as a crucial metabolite that can be readily used for immediate energy requirements, transformed into starch for long-term storage, or employed in diverse biochemical pathways. Glucose is the central molecule of these plants that is commonly produced through photosynthesis and can be converted into starch under certain environmental and physiological circumstances. Saline plants demonstrate the ability to endure osmotic stress and water scarcity by storing excess glucose in the form of starch during drought periods^12^. As plants strive to balance water availability for photosynthesis with energy production needs, sugar levels may fluctuate during the monsoon seasons^13,14^.

Starch, total soluble sugar, and reducing sugar are indispensable carbohydrates in the adaptation to abiotic stress. Total soluble sugar serves as a crucial osmoprotectant, maintaining cellular turgor and osmotic balance under salinity stress conditions^15,16^. Starch acts as a long-term energy reservoir^17^ while reducing sugars provide immediate energy and aid in osmoregulation^10^. Comprehending the mechanisms that underlie the buildup of these carbohydrates is important for revealing the adaptive approaches employed by plants in facing abiotic stress^18^. Comparison of carbohydrate accumulation against the soil quality assessment is crucial for the analysis of soil factors including Electrical Conductivity (EC), Hydrogen Ion Concentration (pH), and Chloride Ion Concentration (Cl)^19^. These parameters play a significant role in understanding their potential correlation with the carbohydrate content, which is essential for their growth^20^.

Water availability has been demonstrated to enhance the accumulation of carbohydrates in plants, especially during periods of the monsoon season^21^. Thus, Fourier Transform Infrared (FT-IR) analysis was specifically executed on samples procured in the monsoon season. FT-IR is an effective analytical method utilised to determine the functional groups present in molecules by measuring their absorption of infrared radiation. This technique enables the analysis of chemical bonds and molecular structures in various samples, such as plant tissues, by quantifying the frequencies of infrared light absorption. Our study utilised FT-IR to analyse the carbohydrate composition and structural characteristics of *Sueda nudiflora*, *Aeluropus lagopoides*, and *Avicennia marina* during the monsoon season. This deliberate decision enabled the exploration of carbohydrate dynamics in saline plants under conditions of increased water availability, offering valuable insights into the mechanisms governing carbohydrate buildup in response to environmental fluctuations. By concentrating on samples obtained during the monsoon season, this study aimed to clarify the role of water availability in driving carbohydrate accumulation in saline plants, aiding in the understanding of their adaptive responses to varying environmental conditions.

By delving deeply into the available literature, sugars were pinpointed and associated based on their distinct spectral patterns in FT-IR spectroscopy. By correlating the observed absorption bands with established functional groups existing in diverse sugar molecules, like hydroxyl, carbonyl, and glycosidic linkages, FT-IR spectroscopy can confirm the structural identification of possible sugars in saline plants. This method, rooted in previous research findings, established a solid framework for characterizing the carbohydrate composition of saline plant species^22,23^, advancing the understanding of their biochemical adaptations to saline environments.

The primary plant species studied were: *Avicennia marina* - A common mangrove species thriving in saline and waterlogged conditions; *Suaeda nudiflora*- A succulent halophyte capable of surviving in high salinity; *Aeluropus lagopides-* A non-succulent halophyte found in saline mudflats. These species are well-adapted to the harsh conditions of the coastal marshes, with physiological and structural adaptations that allow them to thrive in such environments. during three distinct seasons (winter, monsoon, and summer), along with various above-ground plant parts and FT-IR analysis of increased carbohydrate levels in the monsoon period and soil characteristics including EC, pH, and Cl^−^ of winter season. This integrated approach provides a comprehensive insight into the intricate interplay between soil properties and carbohydrate dynamics, thereby enriching the comprehension of the employing of adaptive strategies to cope with abiotic stress and ultimately bolstering environmental resilience.

## Materials and methods

### Study area and plant species

The study examined three plant species, namely *Avicennia marina*, (Forssk.) Vierh. (Avicenniaceae)^24^, *Sueda nudiflora*, (Wild.) Moq. (Chenopodiaceae)^24^, and *Aeluropus lagopides*, (L.) Trin. Ex Thw. (Poaceae)^24^. These plant specimens were sourced from the natural marshy and muddy saline terrain of the Bhavnagar shoreline region, specifically located at coordinates 21°40′37″N, 72°17′08″E. This trial site has a tropical climate characterized by notable seasonal fluctuations. The summer months span from April to June, and are characterized by scorching and arid conditions. The monsoon season, which occurs from July to September, is characterized by substantial precipitation. The winter months, from November to February, are relatively mild, with temperatures. The mean yearly rainfall ranges from around 600 to 800 mm, with the majority occurring during the monsoon period^25^. The seasonal rainfall has a substantial impact on the salinity and water levels in the coastal marshes. The Bhavnagar coastline of the Ghogha trial site is situated along the Gulf of Khambhat and is known for its significant variations in tidal levels^7^. The tides exhibit a semi-diurnal pattern, characterized by the occurrence of two high tides and two low tides within the course of a single day. The tidal range can reach up to 10 m, causing regular flooding of the coastal marshes. This creates a constantly changing and salty environment that is well-suited for the growth of mangroves and halophytes. The soil at the trial site is predominantly saline and muddy, with a substantial clay composition. This soil has a high water retention capacity and is conducive to the growth of plants that can tolerate salt^26^. The salinity of the soil fluctuates in accordance with the tidal cycles, exhibiting elevated levels during arid periods and dilution during the monsoon season. The vegetation in the trial site consists of a combination of halophytes and mangrove species that have adapted to high levels of salinity and occasional flooding^7^. The natural stands of plants were characterized by high salinity and tidal influence. The environmental conditions included regular tidal inundation, which supports the growth and sustenance of these species of plants.

### Sample collection

#### Plant sample collection

Plant samples were collected from the coastline of Ghoha, Bhavnagar. The collection of two halophytes including succulent (*S. nudiflora*) and non-succulent (*A. lagopoides*) and mangrove (*A. marina*) species in replicas of three each following all three seasons over a span of two years. Specifically, the winter collections were done in February and November, the monsoon collections in July and August, and the summer collections in April and May of 2022, and 2023 respectively. The selected specimens represented a range of mature and juvenile stages to provide a comprehensive analysis across different growth phases. The Sampling from natural environment was conducted once per season to capture seasonal variations in plant and soil parameters. The study employed a randomized block design consisting of three replicate blocks to ensure statistical robustness and minimize variability in sampling. Each block contained plots, including individual plant species. Plant and soil samples were collected from plots placed in each block. Soil samples were obtained from the root zones of salt-tolerant plants, including 2 halophytes, and 1 mangrove species of three measurement raplicates to ensure accuracy and reproducibility. Each soil sampling event consisted of 3 samples per individual season. This design guaranteed accurate statistical analysis and reduced variability to a minimum. The study lasted for a duration of two years, specifically from 2022 to 2023, in order to gather comprehensive data across all three seasons. At each sampling event, measurements of height, canopy area, and soil parameters (EC, pH, Cl^−^) were taken and recorded^27^. This was done to account for environmental variability and ensure the accuracy of the data. This approach ensured that the collected data was representative of the natural variability within the population. The stand conditions included semi-dense vegetation with interspersed water channels, indicative of a healthy, well-established coastal ecosystem.

The field-collected plant samples were washed properly with normal tap water followed by RO (Reverse Osmosis) water, to remove all dust particles and intact soil lumps. They were then kept on blotting paper to remove excess water. After that, each plant species’ samples were separated into leaf, stem, and root and weighed followed by drying in a hot air oven at 45 °C. The dried samples were ground into fine powder for the biochemical analysis. These powdered samples were kept in an air-tight condition at room temperature until use. The formal identification of the plant material used in our study was conducted according to the Monograph of Indian Halophytes^28^ authored by Dr. A. J. Joshi, a recognized expert and researcher in the field of coastal habitats. A Voucher specimen number BKNMU213, BKNMU214 and BKNMU215 and deposition numbers DLS455, DLS456, and DLS457 of the material have been securely deposited at the Department of Life Sciences, Bhakta Kavi Narsinh Mehta University. These plant samples were collected in accordance with WHO guidelines^27^.

At the site, during the collection of plant species, detailed measurements of plants’ height and canopy area were recorded to provide insights into growth characteristics (Table 1). The average height and canopy area were:

**Table 1:** Height and canopy area of mangrove and halophytic plant species.

Plant species	Height in cm (mean)	Area in cm^2^ (mean)
*Aeluropus lagopoidis*	26.6	13,665.5
*Suaeda nudiflora*	30.12	8804.5
*Avicennia marina*	77.5	10,409
Standard deviation	28.42553078	2476.843875

#### Soil sample collection

Root zone soil samples were collected from the coastline of Bhavnagar, Gujarat at a depth of 0–20 cm top soil layer from each plant species (as listed above) with the help of a soil auger. Samples of soils were taken for study and brought to the laboratory. Initially, samples were air-dried and crushed to pass through a 2 mm sieve and stored at room temperature by sealing them in zip-lock polythene bags. The study employed a randomized block design with three replicate blocks to ensure statistical robustness and minimize variability in sampling^19^.

### Carbohydrate estimation

#### Determination of reducing sugar

A 70% methanolic extract was obtained by processing 100mg of plant materials. After the methanol evaporated, the remaining substances were dissolved in distilled water. An alcohol-free extract was taken, measuring 0.2ml. The extract was subjected to treatment with an alkaline solution of DNS (3,5-Dinitrosalicylic Acid), and the resulting mixture was heated for 5min, resulting in the formation of 3-amino-5-nitrosalicyclic acid. After the cooling process, the measurement of absorbance was performed at a wavelength of 510nm^29^. The quantification of sugar content reduction was performed by utilizing a standard graph derived from a glucose solution with a concentration of 100μgml^-1^.

#### Determination of total soluble sugar

A plant sample weighing 100 mg was subjected to hydrolysis using 2.5 N hydrochloric acid (HCl). Following the neutralization process, the liquid portion (supernatants) was gathered, and a 0.1 ml sample was examined using an anthrone reagent. The absorbance at a wavelength of 630 nm was determined, and the concentration of total soluble sugar was calculated using a standard graph^30^.

#### Determination of total starch content

The plant sample, weighing 100 mg, was thoroughly mixed with a hot methanol solution containing 70% methanol. Following centrifugation, the residues were cleansed, desiccated, and subjected to 52% perchloric acid treatment. Supernatants were gathered, and the amount of glucose was measured using an anthrone reagent^31^. The total starch content was determined by multiplying it with a factor of 0.9.

### Soil analysis

Root zone soil samples were processed by preparing a soil solution with a 1:2 ratio, which was then agitated on a shaker for 24 h. The resulting mixture went through the filtration (Whatman 44), producing a clear extract. Subsequently, the clear extract was utilized for the measurement of EC, pH, and Cl^−^ measurement^19^. The estimation of Cl^−^ content was carried out using potassium chromate as an indicator, titrating against 0.1 N AgNO_3_. All these analyses were conducted using 100 g of soil for each set of measurements.

### FT-IR analysis

FT-IR analysis was implemented to identify the presence of functional groups in the above-ground parts of mangrove and halophyte. For the FT-IR analysis, Leaves of *Sueda nudiflora*, *Aeluropus lagopoides*, and *Avicennia marina* were collected specifically during the monsoon season from the Bhavnagar coastline. Upon collection, leaves were carefully washed with distilled water to remove surface contaminants and dried at 40 °C until reaching a constant weight. The dried leaves were then ground into a fine powder using a mortar and pestle. Approximately 2 mg of each powdered leaf sample was mixed with KBr and ground thoroughly to ensure homogeneity. Subsequently, the powdered mixture was compressed into pellets using a hydraulic press at 10 tons/cm² pressure. FT-IR spectra of the leaf samples were acquired using a spectrometer equipped with an ATR accessory, scanning from 4000 to 400 cm^−¹^ with a resolution of 4 cm^−¹^ using an FT-IR instrument (PerkinElmer Spectrum 65 series, USA). Blank KBr pellets were used for baseline measurements, and each leaf sample was analyzed in triplicate to ensure reproducibility^32^. The acquired spectral data were analyzed to identify characteristic absorption bands corresponding to major biochemical constituents focusing on carbohydrates present in the leaves of these plant species during the monsoon season.

### Statistical analysis

The study employed statistical analyses to uncover significant relationships within the dataset. The use of hierarchical analysis enabled the identification of patterns, while correlation matrix analysis was used to examine the associations between variables. PCA was utilized to decrease dimensionality and extract essential features. In addition, ANOVA was utilized to examine variations between groups, offering a thorough comprehension of the statistical attributes of the sample. In addition, the Partial Least Squares (PLS) regression was employed to examine the relationships between soil and carbohydrate variables. K-means clustering was also used to identify separate clusters within the FT-IR dataset, which helped to better understand the underlying structure and patterns of sugars.

The study employed a comprehensive set of statistical analyses to uncover significant relationships within the dataset. Hierarchical analysis was used to identify similarities in sugar accumulation patterns among different plant parts, while correlation matrix analysis examined how carbohydrate accumulation patterns relate to halophytes’ and mangroves’ metabolic responses under varying environmental conditions. Principal Component Analysis (PCA) reduced dimensionality and identified primary components of data variance associated with seasonal sugar accumulation in various plant parts. In addition, ANOVA assessed the statistical significance of variations in total starch, total soluble sugar, and reducing sugar compositions across different plant parts and seasons. Partial Least Squares (PLS) regression provided insights into the relationships between predictor variables (total carbohydrates), and response variables (EC, pH, and Cl^−^). K-means clustering of FT-IR data revealed two distinct groupings, highlighting the fundamental composition of the dataset and separating it into distinct spectral characteristics. All analyses were conducted using OriginPro software, offering a thorough understanding of the statistical attributes of the sample and revealing underlying patterns in sugar dynamics influenced by changing variables.

## Results

### Carbohydrate accumulation pattern in saline plants

**Fig. 1:**
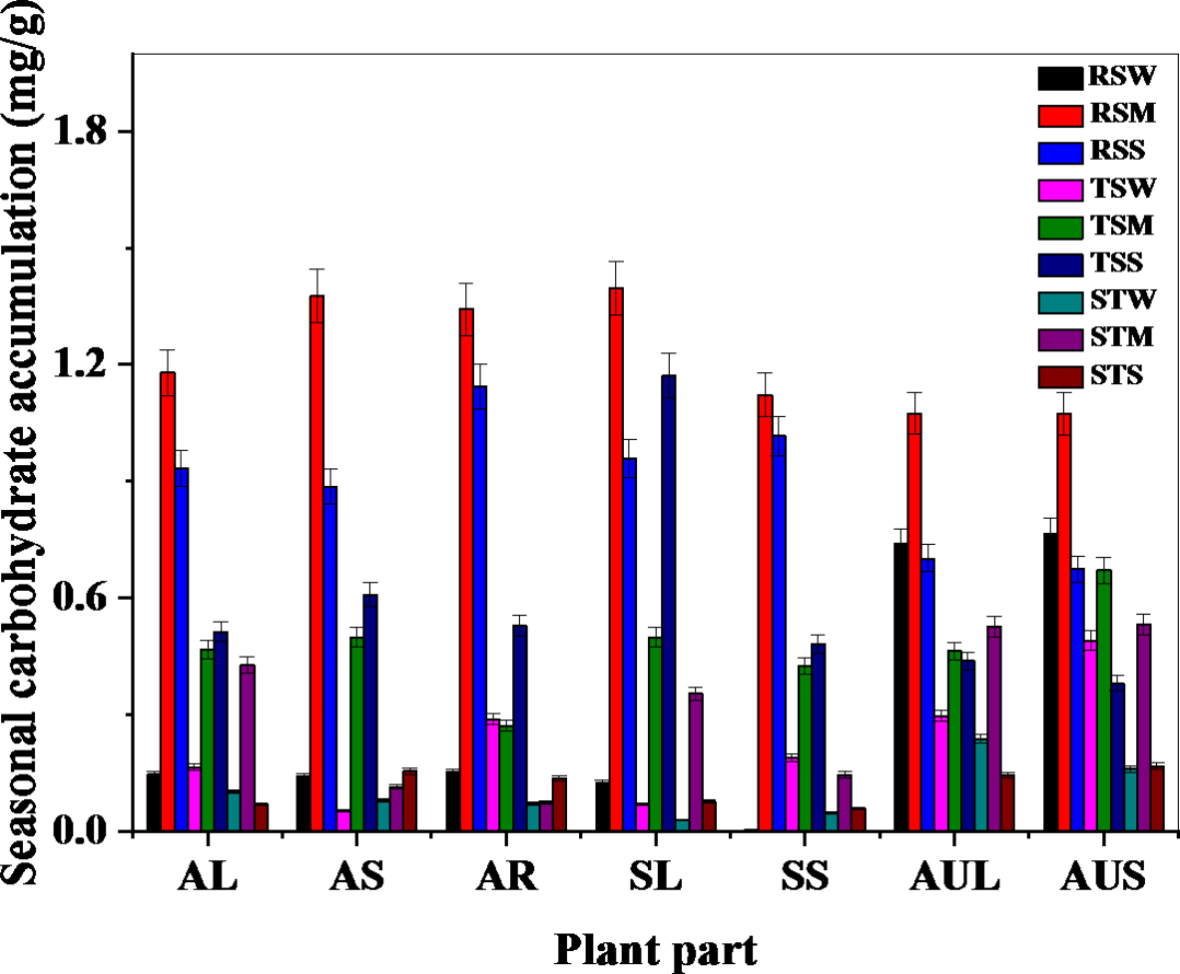
Seasonal carbohydrate accumulation in above-ground parts of saline plants.

The patterns of carbohydrate accumulation in *Avicennia marina*, *Aeluropus lagopoides*, and *Sueda nudiflora* demonstrate significant seasonal variations (Fig. 1), indicating the adaptation of these plants to fluctuating environmental conditions in various species and plant parts.

#### Succulent halophyte - *sueda nudiflora*

Seasonal variations in the levels of reducing sugar and total soluble sugar were prominently observed in *S. nudiflora*. The presence of the monsoon season notably influenced the decline in sugar content, reaching its zenith at 1.3971 mg/g, signifying an enhancement in metabolic processes and photosynthetic activity due to favorable environmental conditions. Similarly, the total quantity of soluble sugars followed a similar trend, peaking at 0.4992 mg/g in the monsoon season. These observed patterns suggest the presence of efficient mechanisms in the plant species for maintaining osmotic equilibrium and coping with various stressors including salinity and seasonal variations. More precisely, these adjustments refer to changes regarding physiological processes in carbohydrate metabolism to regulate osmotic balance. These adjustments can be aided by increased metabolic processes and photosynthetic activity, particularly during favorable periods like the monsoon season.

#### Non-succulent halophyte - *Aeluropus lagopoides*

On the contrary, *A. lagopoides* displayed a consistent decrease in sugar levels over the duration of the year, indicating a steady metabolic rate. During the monsoon season, there were marginal enhancements in the reduction of sugar content (1.0750 mg/g) and total soluble sugar (0.4634 mg/g), underscoring the species’ efficient regulation of sugars in saline habitats.

#### Mangrove - *Avicennia marina*

The pneumatophores of *A. marina* exhibited remarkable seasonal variations. In the period of monsoon, a notable rise in the concentrations of reducing sugar and total soluble sugar within the pneumatophores was evident. Particularly, the diminishing sugar content hit 1.1796 mg/g, with the total soluble sugar content registering at 0.4670 mg/g. The roots manifested the highest level of total starch, a vital energy storage component, during the monsoon season. This suggests the presence of deliberate adaptations to address the escalated energy requirements during this period. The observed trends underscore the critical significance of pneumatophores in the metabolic adaptability of *A. marina* to diverse environmental conditions in the intertidal region, thereby bolstering the overall resilience of mangrove ecosystems against disturbances.

The distinctive patterns of sugar accumulation in halophytes and mangroves underscore the adaptation strategies crucial for their viability in saline habitats. The findings shed light on the intricate interplay between seasonal variations and plant responses, augmenting our understanding of the adaptability and survival mechanisms utilized by these plants in challenging environments.

(Note: AL: *A. marina* leaf, AS: *A. marina* stem, AR: *A. marina* pneumatophore, SL: *S. nudiflora* leaf, SS: *S. nudiflora* stem, AUL: *A. lagopoides* leaf, AUS: *A. lagopoides* stem, STS: starch accumulation in summer, STM: starch accumulation in monsoon, STW: starch accumulation in winter, TSS: total soluble sugar accumulation in summer, TSM: total soluble sugar accumulation in monsoon, TSW: total soluble sugar accumulation in winter, RSS: Reducing sugar accumulation in summer, RSM: Reducing sugar accumulation in monsoon, RSW: Reducing sugar accumulation in winter.)

### FT-IR analysis

FT-IR spectroscopy provides a sturdy technique for assessing the physiochemical attributes of carbohydrates. The FT-IR spectra of raw specimens from *A. marina*, *S. nudiflora*, and *A. lagopoides* exhibited carbohydrate bands spanning from 3500 cm^− 1^ to 426 cm^− 1^ (Supplementary Fig. 1A). The appearance of peaks at 1649 cm^− 1^ and 3100 cm^− 1^ indicates the crystallization of water within the specimen. The spike at 1649 cm^− 1^ is due to the bending vibrations of water molecules, while the spike at 3100 cm^− 1^ is associated with the stretching vibrations of O-H bonds in water (Supplementary Fig. 1A,B ,C).

The FT-IR spectra of unrefined polysaccharides displayed carbohydrate bands within the range of 3441.01 cm^− 1^ to 451.34 cm^− 1^. A peak at 3444.87 cm^− 1^ specifically identified the hydroxyl group, while the methylene group was distinctly observed at 2929.82 cm^− 1^ (Supplementary Fig. 1C). Moreover, the existence of anionic carbohydrate groups was verified by a peak at 1649.14 cm^− 1^, along with the identification of an α-CH_3_ group at 1255.66 cm^− 1^. These results point towards the presence of Rhamnose sugar in *S. nudiflora* (Supplementary Fig. 1B).

Furthermore, the FT-IR spectrum of raffinose in *A. lagopoides* displayed prominent peaks at wavenumbers 935 cm^− 1^, 996 cm^− 1^, and 1152 cm^− 1^, suggesting the existence of alpha 1,6-glycosidic bonds within the sugar units of the compound (Supplementary Fig. 1A). These peaks seem to deviate from the expected structural composition of raffinose, featuring a trisaccharide unit connected by alpha 1,6-glycosidic bonds. The detection of fructan linkages within the sugar molecule of *A. marina* and *S. nudiflora* was highlighted by the peak at 1050 cm^− 1^ indicating the resemblance of the FT-IR spectrum with inulin (Supplementary Fig. 1B,1 C). Fructans are intricate carbohydrates primarily composed of fructose units connected via beta (2→1) glycosidic bonds. This particular peak serves as a pivotal indicator for discerning the fructan structure of inulin using FTIR spectroscopy. Moreover, the FT-IR spectrum of trehalose indicated unique peaks that aligned with its structural attributes, affirming its presence in *A. marina*, *S. nudiflora*, and *A. lagopoides*. Peaks at 3500 cm^−1^ and 1680 cm^− 1^ corresponded to the stretching vibrations of O-H bonds and the bending vibrations of water molecules (H-O-H bending), respectively, providing proof of water molecules in the sample. Moreover, the peaks seen at 994 cm^− 1^ and 954 cm^− 1^ point towards the presence of glycosidic linkages in the trehalose molecule. The peak at 994 cm^− 1^ is a key feature of the stretching of the C-O bond in the glycosidic linkage, with the peak at 954 cm^− 1^ likely reflecting the stretching vibrations of the C-O-C bond. Also, the peaks detected at 998 cm^− 1^ and 956 cm^− 1^ were designated as the symmetric and antisymmetric stretching vibrations, respectively, suggesting the presence of alpha-(1–1)-glycosidic bonds in trehalose (Supplementary Fig. 1).

The FT-IR spectra of glucose in *A. marina*, *S. nudiflora*, and *A. lagopoides*, as well as sucrose in *A. marina* and *A. lagopoides*, shown in the supplementary file display the unique peaks suggestive of their particular structural traits. The peaks recognized at 1077 cm^− 1^ and 1062 cm^− 1^ are related to the stretching vibrations of C-O bonds, implying the existence of carbonyl groups within the molecules. Furthermore, peaks at 1268cm^− 1^, 1124 cm^− 1^, and 915 cm^− 1^ reflect vibrations connected to C-C-H, O-C-H, and C-O-H bonds, correspondingly, showcasing the primary structure and functional groups of the sugars. In addition, peaks at 1457 cm^− 1^, 2946 cm^− 1^, and 1365 cm^− 1^ are indicative of the asymmetric and symmetric stretching vibrations of CH_2_ groups, which are commonly present in carbohydrates. Also, the peaks at 777 cm^− 1^ and 915 cm^− 1^ point to C-H deformation. Finally, peaks observed at 524 cm^− 1^ and 426 cm^− 1^ correspond to C-C-O and C-C-C vibrations, respectively, reflecting the fundamental structure of the sugars. In summary, these distinct peaks offer valuable insights for differentiating and classifying glucose and sucrose based on their molecular compositions and structural attributes, providing essential information for identification purposes.

### Soil analysis

**Fig. 2:**
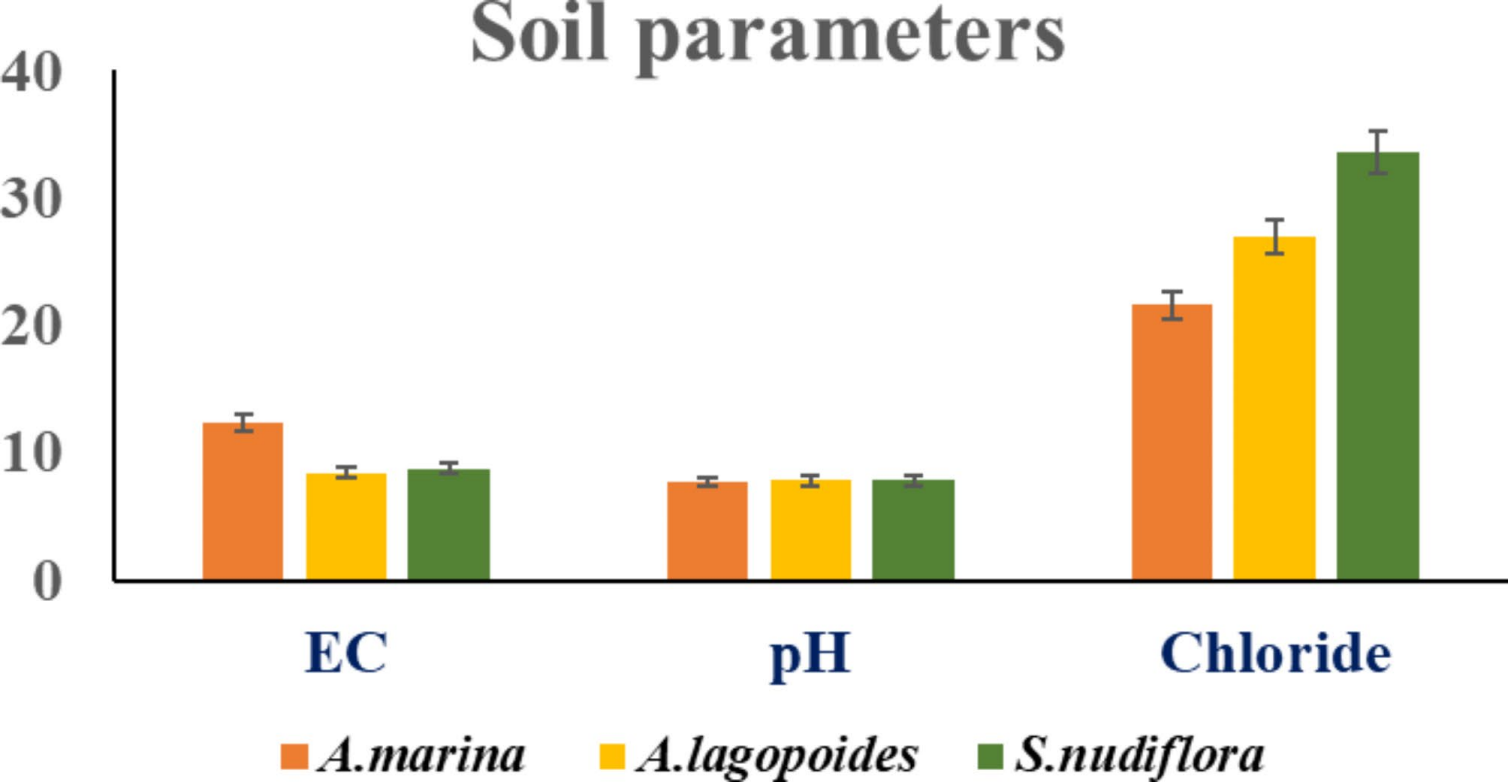
Soil parameters of underground parts of saline plants.

The soil properties of *A. marina*, *A. lagopoides*, and *S. nudiflora* are presented in the graph with error bars (Fig.2). Electrical conductivity determines the ability of solutions to carry an electric current. EC is employed in soil science to quantify the concentration of dissolved salts and ions. Elevated EC values indicate elevated concentrations of dissolved salts. pH is a measure of the acidity or alkalinity of a solution that can impact on nutrient availability, microbial activity, and plant health. The presence of chloride ions in soil is crucial for plant nutrition, although elevated levels can lead to soil salinity. Monitoring chloride levels aids in evaluating soil fertility and controlling irrigation to prevent plant toxicity. These differences in EC, pH, and Cl^−^ indicates the adaptations made by these salt-tolerant plants to their environments. Higher EC values in root-associated soils of *A. marina* (12.37mS^−1^) than in *A. lagopoides* (8.409mS^−1^) and *S. nudiflora* (8.801mS^−1^) imply different levels of tolerance to soil salinity among them. The carbohydrate synthesis relationship with the soil can be seen from this figure as well as chloride concentration levels which may have enabled the adaptation of *A. lagopoides* to saline environments (27.03meqL^−1^). Conversely, different preferences might exist for certain types of soils by *S. nudiflora* which has got least Chloride value than any other plants studied here (33 0.64meqL^−1^). The similarity between slightly acidic and neutral soil pH ranges of 7.74–7.85 suggests that these two species exhibit almost similar preferences towards acidity or alkalinity within their growth media so maybe they employ different metabolic strategies because total carbohydrates content is highest in *A. lagopoides* (1.19mgg^−1^). This research therefore reveals the interactions between soil and saline plants resulting in influencing carbohydrate accumulation revealing some ecological implications too significant for agriculture development at a large-scale level.

### Statistical analysis

#### Hierarchical analysis

**Fig. 3:**
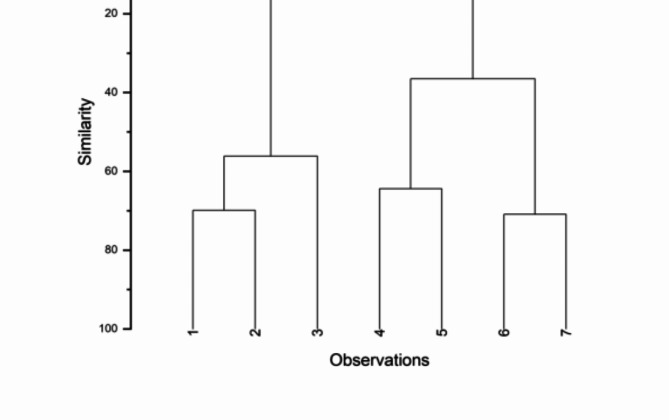
Hierarchical analysis of sugar accumulation pattern similarities in saline plant parts. 1- *A. marina* leaf, 2- *A. marina* stem, 3- *A. marina* root, 4- *S. nudiflora* leaf, 5- *S. nudiflora* stem, 6- *A. lagopoides* leaf, 7- *A. lagopoides* stem.

Hierarchical analysis was used to find similarities in sugar accumulation patterns in plant parts. Pearson correlation distance was used to measure the similarity between clusters. Figure 3 shows six clusters identified using the average group method and Pearson’s correlation distance.

Based on accumulation and composition similarity, clusters were formed that produced a dendrogram with six major clusters and sample similarity percentages. *A. marina* leaf and stem shared 75.43% similarity in the first cluster. The second cluster had *A. marina* root that was 65.28% similar to the first. The fourth cluster *A. lagopoides* leaf and stem showed 48.36% similarity, while the third cluster *S. nudiflora* leaf and stem showed 67.05%. The fifth cluster had the lowest similarities (35.48%) to the third and fourth clusters, indicating large carbohydrate accumulation differences between succulent/non-succulent halophytic plants. However, no similarities were found between mangrove and sixth cluster halophytes.

#### Analysis of correlation

**Fig. 4:**
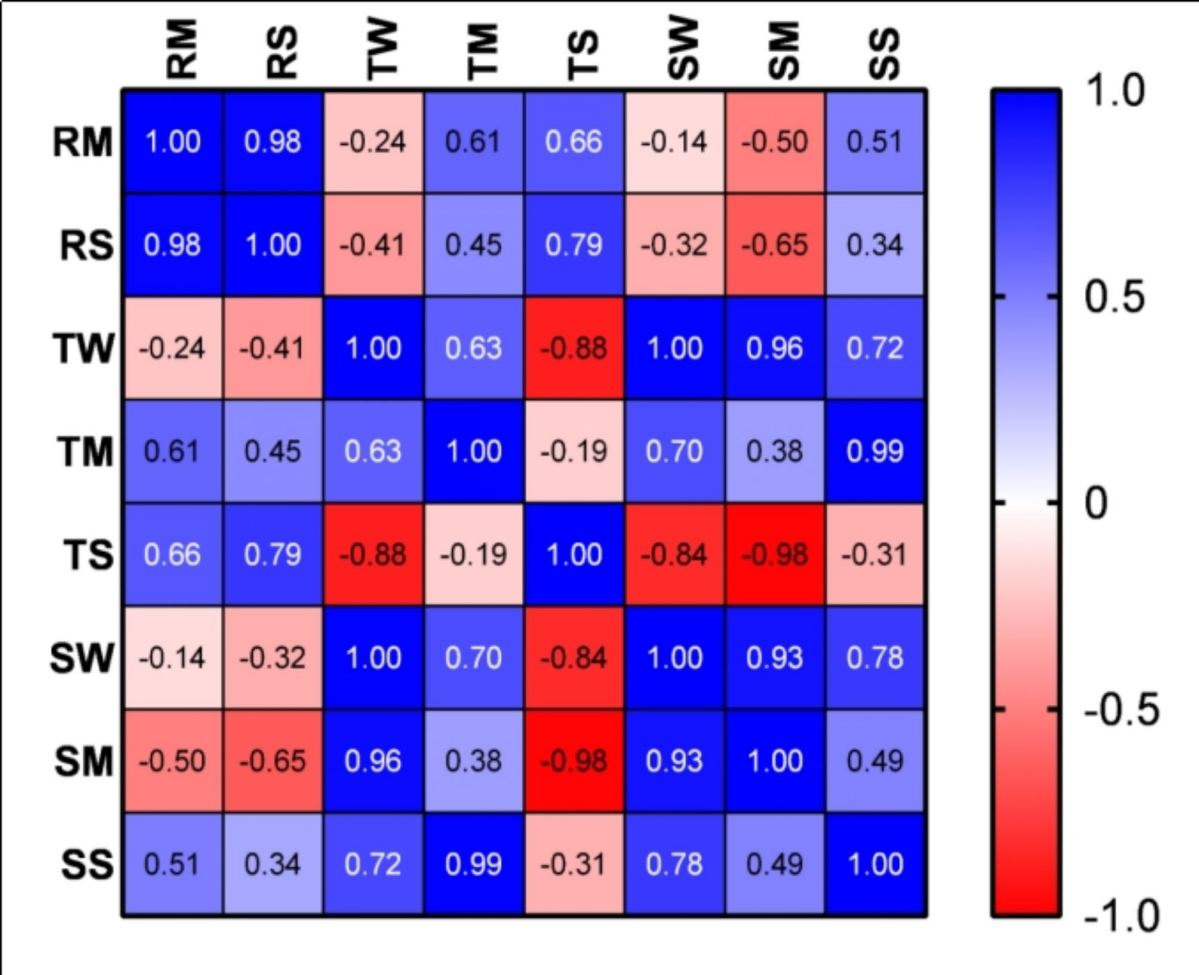
Pearson’s correlation matrix analysis of sugar accumulation in different seasons.

(Note: SS: starch accumulation in summer, SM: starch accumulation in monsoon, SW: starch accumulation in winter, TS: total soluble sugar accumulation in summer, TM: total soluble sugar accumulation in monsoon, TW: total soluble sugar accumulation in winter, RS: Reducing sugar accumulation in summer, RM: Reducing sugar accumulation in monsoon.)

The correlation matrix in Fig. 4 shows how our study of carbohydrate accumulation patterns relates to halophytes’ and mangroves’ metabolic responses under different environmental conditions. A strong positive correlation (0.98) was found between monsoonal mangrove samples’ reducing sugar content and summer halophytes. Due to common factors affecting levels in both types across seasons, these species may have similar sugar accumulation patterns.

A notable finding is that the succulent halophyte *S. nudiflora*’s soluble sugar content moderately positively correlates during monsoon and summer. This suggests a seasonal pattern of sugar accumulation, possibly due to similar responses to water or metabolic activity. The non-succulent halophytes *A. lagopoides* (summer) and *S. nudiflora* (monsoon) have a moderate positive correlation (0.49) in stem starch levels. This correlation suggests that salinity may influence these plants’ environmental adaptations.

Total soluble sugar in halophytes in winter and mangrove starch in summer are moderately negatively correlated (−0.88). This suggests a trade-off between sugar types as organisms may allocate resources differently to survive under certain conditions. Another intriguing finding is the strong negative correlation (−0.84) between mangrove total starch during the monsoon and succulent halophytes’ sugar levels in winter. These two plant species respond differently to environmental signals, possibly due to salinity differences. It also implies that they store energy differently year-round.

Studies of these connections help us understand the complex metabolic changes these plants undergo and the surrounding changes. Understanding how carbohydrates accumulate helps plants adapt to and survive stress. Future research and conservation efforts to protect these plants and their habitats require this knowledge.

#### Principal Component Analysis (PCA)

**Fig. 5:**
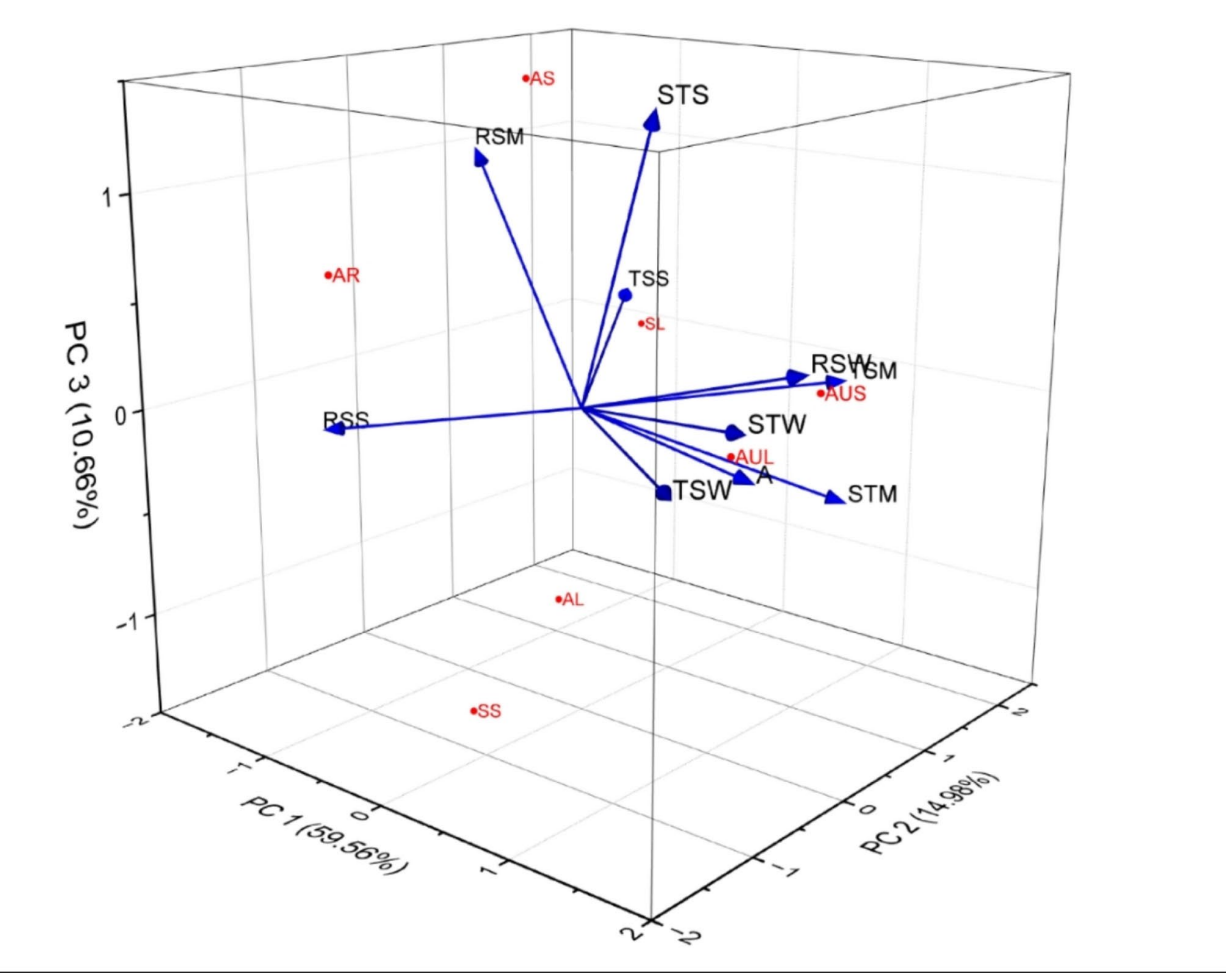
Principal Component Analysis of sugar accumulation with seasonal variation in saline plant parts.

Principal Component Analysis (PCA) reduced dimensionality and identified primary components of data variances (Fig. 5). Eigenvalues show the extent to which each principal component explains variances, with higher eigenvalues explaining more variance. The primary principal component has the highest eigenvalue of 5.95623, explaining 59.56% of the variance. PC2, with the second-highest eigenvalue of 1.49768, adds 14.98% variance. PC3 explains 10.66% of the total variation, with subsequent components explaining smaller amounts. The initial three principal components explain 85.20% of the variance. The fourth principal component increases explanatory capacity to 92.26%. The top five factors explain 97.26% of the variance. These findings highlight the importance of PC1 in revealing the dataset’s intrinsic framework for further data analysis.

#### Analysis of variance

Research was conducted to take a peek into the total starch, total soluble sugar, and reducing sugar composition in different plant parts and seasons. Single-factor ANOVA was used for the statistical analysis of the data to determine its significance. Succulent plants showed lower sugar levels than mangrove plants, with significant differences in sugar accumulation (*p*-value: 0.00035.829). A statistical difference of note was found when mangroves were compared to non-succulent plant species (*p*-value: 0.00008879). Non-succulent plants were seen to have lower sugar content. The sugar content of succulent species was found to be higher than that of non-succulent plant species (*p*-value: 0.019901823). Significant differences in the mangrove and halophyte groups were also noted in the accumulation of sugars. With a *p*-value of 0.000382557, the halophytes showed increased sugar levels. These results show the way plant category and seasonal changes affect sugar levels and offer important insights into the physiological adaptability of plants to saline conditions.

#### Partial Least Squares (PLS) analysis

**Fig. 6:**
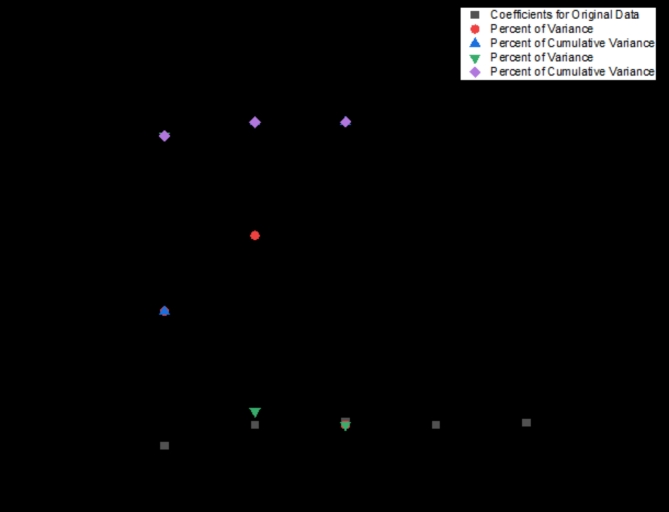
Partial Least Squares regression in total carbohydrates-based individualized estimation.

The Partial Least Squares (PLS) model imparts insights into the connections between the predictor variables and the response variable (Fig. 6). Functioning in the capacity of a reference point, the intercept is around − 6.87, demonstrating the expected value of the response variable with all predictors at zero. The coefficient related to EC is −0.03, implying a minor negative correlation, although it lacks statistical significance. With a coefficient of 1.03, the pH coefficient hints at a constructive effect on the response variable, illustrating significant statistical importance (*p* < 0.05). Despite Cl^−^ having a coefficient of −0.03, additional data is necessary to determine its statistical significance conclusively. Total Carbohydrates displays a positive and statistically significant relationship with the response variable, showing a coefficient of 0.78. In general, the model imparts valuable insights regarding the influence of these predictors on the response variable, emphasizing the significance and directionality of the coefficients. A thorough examination of the statistical significance of all coefficients and careful adherence to model assumptions are essential for a thorough interpretation.

#### K means clustering

**Fig. 7:**
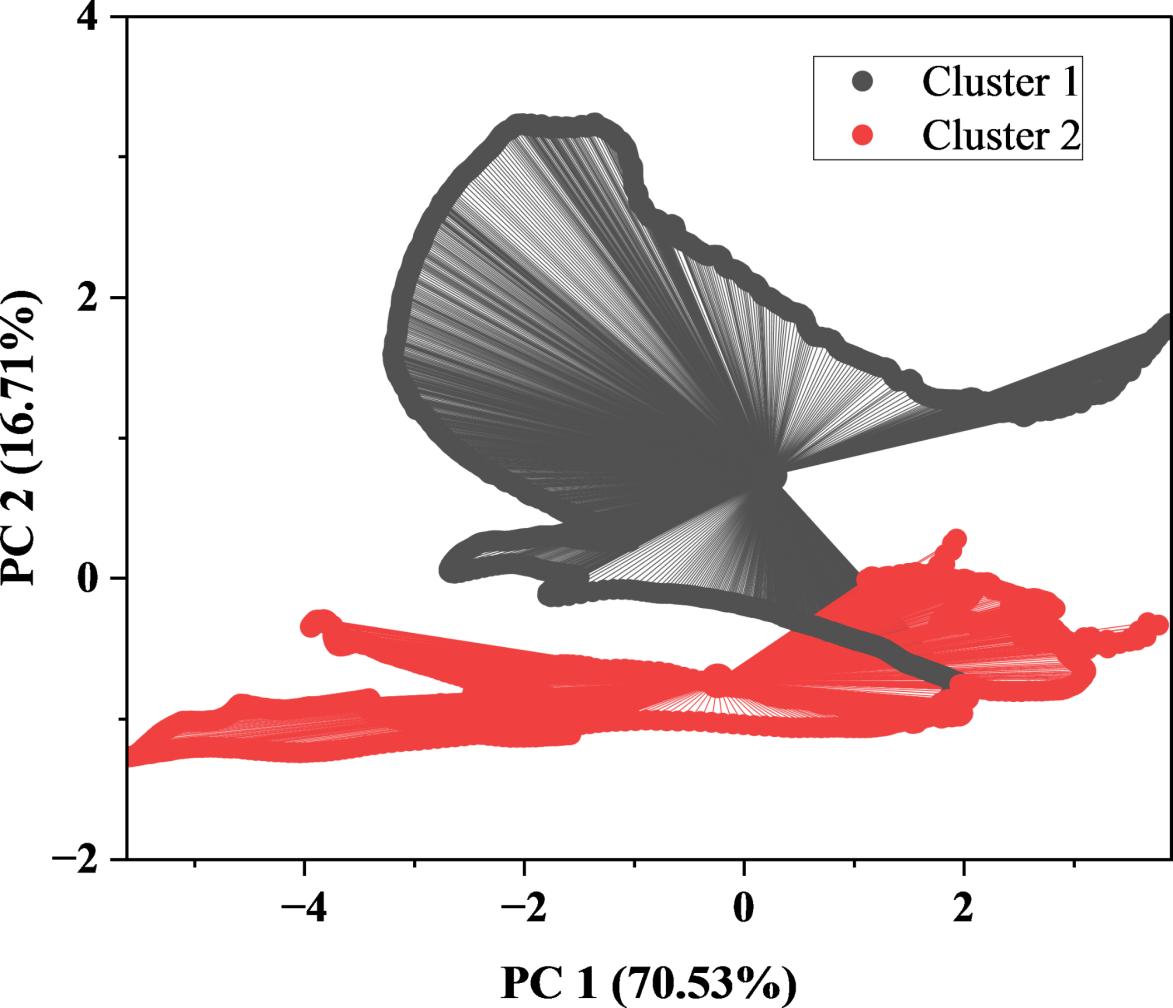
K means cluster analysis in FT-IR data.

K-means clustering on FT-IR data revealed two sets (Fig. 7). Clusters 1 and 2 had centers at 4000 cm^− 1^ and 400 cm^− 1^, respectively. After convergence, the final cluster centers shifted to 3100 cm^− 1^ for Cluster 1 and 1299.5 cm^− 1^ for Cluster 2, with spectral intensities at these wavenumbers varying for each cluster. The cluster summary statistics showed similar numbers of observations, sum of squares, and average distances within each cluster. The ultimate cluster centers were 1800.538 apart, indicating a significant separation. The score plot from the principal components analysis showed clear differences between groups along PC1 and PC2, explaining 70.53 and 16.71% of variability, respectively. These findings indicate the successful separation of FT-IR data into two distinct groupings with different spectral characteristics, revealing the dataset’s fundamental composition.

**Fig. 8:**
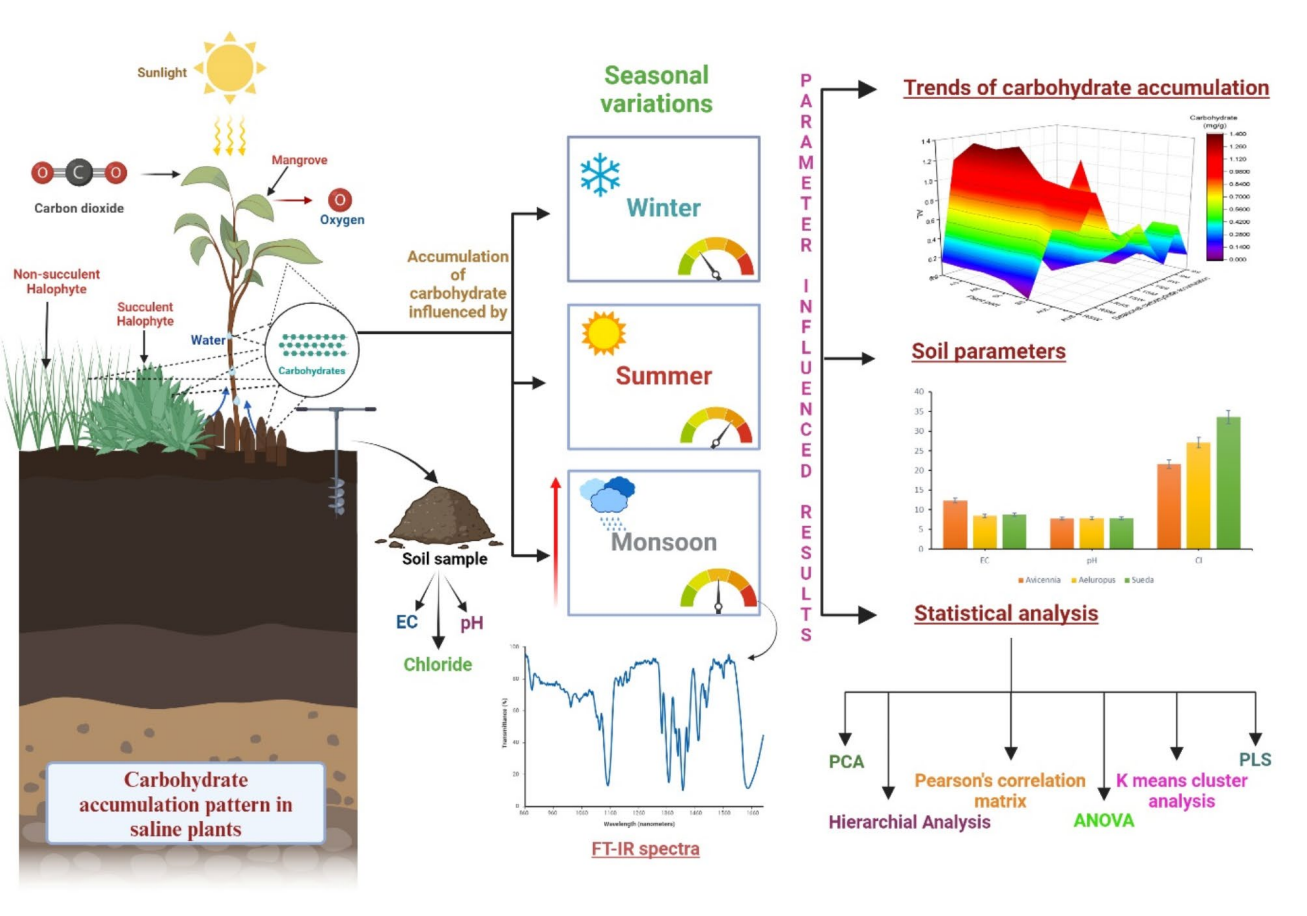
Evaluation of carbohydrate accumulation trends among mangrove and halophytic plant species across three distinct seasons.

In summary, as illustrated in Fig. 8, the study found notable seasonal fluctuations in sugar levels among distinct halophytic and mangrove species across different above ground parts throughout the entire year. FT-IR analysis was conducted to examine the elevated levels of carbohydrates during the monsoon period, as well as the soil characteristics of the winter season, including electrical conductivity (EC), pH, and chloride ion concentration (Cl^-^). This integrated approach offers a thorough understanding of the complex relationship between soil properties and carbohydrate dynamics. The multivariate analyses revealed distinct patterns in the accumulation of sugar and its correlation with soil characteristics, indicating complex interactions. These findings emphasise the significance of carbohydrate metabolism in the ability of coastal plants to tolerate stress and allocate resources.

In summary, as illustrated in Fig. 8, the study found notable seasonal fluctuations in sugar levels across different above-ground parts of distinct halophyte and mangrove species throughout the entire year. FT-IR analysis was conducted to examine the elevated levels of carbohydrates during the monsoon period, as well as the soil characteristics of the winter season, including electrical conductivity (EC), pH, and chloride ion concentration (Cl^−^). This integrated approach offers a thorough understanding of the complex relationship between soil properties and carbohydrate dynamics. The multivariate analyses revealed distinct patterns of sugar accumulation and its correlation with soil characteristics, indicating complex interactions. These findings emphasise the significance of carbohydrate metabolism in the ability of coastal plants to tolerate stress and allocate resources.

## Discussion

*Avicennia marina* is a mangrove species that plays a crucial role in stabilizing coastlines, mitigating erosion, and serving as a habitat for both marine and terrestrial species. The dense root systems of this organism effectively capture sediments and offer protection^[1.2]^. *Sueda nudiflora* is a halophyte that can tolerate high levels of salt. It is commonly found in saline environments and plays a role in stabilizing soil and enhancing fertility. It captures soluble sugars and glucose, serving as osmoprotectants and supplying instant energy^4,5^. *Aeluropus lagopoides* exhibits adaptations that enable it to thrive in highly saline environments, effectively stabilizing soil, mitigating erosion, and enhancing soil structure. Its primary function is to store polysaccharides, such as inulin and cellulose, which are crucial for energy storage and stress resistance^4,7,15^.

The presented results emphasize significant discoveries regarding carbohydrate metabolism in various plant parts and species being investigated. Our observations revealed clear patterns in total starch, soluble sugars, and reducing sugar levels in different seasons and plant parts. This highlights the impact of both seasonal changes and species-specific characteristics on the accumulation of carbohydrates. These observations are crucial for comprehending how these plants regulate osmotic equilibrium when faced with hyperosmotic stress, demonstrating their adaptive tactics in response to environmental fluctuations. During moments of hyperosmotic stress, osmolytes are vital in the adjustment of osmotic balance. In response to saline stress, sugars are produced as a metabolic response, serving as osmolytes^33^.

Examining the trends of carbohydrate accumulation in *A. marina*, *A. lagopoides*, and *S. nudiflora* may uncover valuable information on the adaptive mechanisms of these plants to different environmental conditions and seasonal variations. *S. nudiflora*, a halophytic species, exhibited a notable increase in both reducing sugar and total soluble sugar levels during the monsoon season, likely due to conducive conditions stimulating heightened metabolic processes and photosynthesis. Conversely, *A. lagopoides* showed a consistent, gradual decrease in sugar levels throughout the year, indicating a stable metabolic rate unaffected by seasonal changes^4^. *A. marina*, a mangrove plant, displayed significant seasonal variations in sugar accumulation, particularly in pneumatophores during the monsoon season. Studies suggest that roots displayed markedly elevated levels of reducing sugar and total soluble sugar, underscoring the vital role of pneumatophores in enabling mangrove ecosystems to thrive in harsh intertidal environments^2^.

The findings acquired from FT-IR spectroscopy give important details on the physiochemical properties and structural attributes of carbohydrates harvested from the aerial parts of mangrove and halophyte species. The presence of distinct bands in the FT-IR spectra of both crude and purified samples indicates the diverse carbohydrate composition within these plant species. The characteristic peaks in the spectra were compared to the spectra of known glycoconjugates to identify the glycoconjugates present in the samples^34^. Functional groups like hydroxyl and methylene indicate distinctive raw polysaccharides in specimens^22^. Additionally, the detection of anionic carbohydrate groups and α-CH_3_ groups further bolsters the identification of specific sugars, like Rhamnose, in the studied *S. nudiflora* species. These peaks align with previous reports by Seedevi et al.^23^ on rhamnose-enriched polysaccharides in *Grateloupia lithophila* on α-L-Rhmnose in *Penicillium chrysogenum* with similar wavenumbers^18^.

The identification of distinctive peaks in the FT-IR spectra of raffinose, inulin, trehalose, glucose, and sucrose aids in understanding their structural compositions and supports the distinguishing of different sugars. For example, the presence of alpha 1,6-glycosidic bonds in raffinose and fructan linkages in inulin is confirmed by distinct peaks in their spectra^35^. Similarly, trehalose manifests unique peaks that are indicative of its structural attributes, including glycosidic linkages and alpha-(1–1)-glycosidic bonds^22^. The FT-IR spectra of glucose and sucrose also exhibit specific peaks indicative of their structural compositions, enabling differentiation based on molecular arrangements and structural characteristics^36^.

The FT-IR spectroscopy results significantly contribute to our comprehension of the carbohydrate makeup and structural variability observed in mangrove and halophyte species. For example, Trehalose represents a non-reducing disaccharide composed of glucose, known for its high solubility and chemical stability, thereby facilitating its utilization in cellular metabolism even under high concentrations^37^, thereby playing a crucial role in abiotic stress resilience. Trehalose functions to safeguard cellular membranes and proteins^38^, leading to current investigations into plant genetic modification due to the effectiveness of the trehalose synthesis gene. Plants articulating these genes have displayed heightened resilience to various abiotic stressors. In times of abiotic stress, trehalose serves as an immediate energy source, influencing the regulation of plant starch metabolism as well^39^.

Conversely, fructans are known to store carbohydrates in numerous plant species^38^. Plants store fructans within their vacuoles, helping them to endure abiotic pressures^40^. The precise tolerance mechanism attributed to fructan accumulation remains unclear. Nonetheless, there is a theory that during stressful conditions, fructans may have interactions with glutathione, potentially participating in the ascorbate cycle or cell signaling pathways^41^. Rhamnose, as reported by Mehta and Vyas^7^, is discernible in halophytes, particularly in plants of the Amaranthaceae family. In *S. nudiflora*, Rhamnose is speculated to be significant in the creation of pectic polymers and cell wall glycoproteins^42^. Through spectral analysis, *A. lagopoides* emerged as the sole known origin of Raffinose, derived from sucrose. These compounds, categorized as compatible solutes, are integral to stress tolerance mechanisms, while also displaying antioxidant properties and contributing to carbon allocation strategies and potential stress response signaling^43^. These insights could enrich our understanding of plant adaptation strategies in saline environments.

Through a hierarchical analysis, valuable insights were obtained into the relationships among different plant components^44^ and their carbohydrate accumulation. This analytical approach facilitated the recognition of both commonalities and distinctions among these components. Examination of carbohydrate levels in various plant segments reveals distinct accumulation patterns in succulent and non-succulent halophytic plants. Correlation analysis revealed significant positive associations between diverse sugar accumulation patterns in halophytes and mangroves, indicating mutual responses to environmental stimuli. Intriguing relationships exist among different sugar types in coping with environmental challenges, possibly implying trade-offs as organisms adapt and strive for survival^45^.

Principal Component Analysis (PCA) was pivotal in minimizing data dimensions, with PC1 outlining a considerable portion of the total variability, emphasizing its crucial role in grasping significant disparities. Insight into variance distribution provides a crucial understanding of dataset structure and dimensionality^46^. Single-factor ANOVA analysis by Li et al.^47^ revealed notable variations in sugar accumulation patterns, shedding light on the impact of different factors on sugar levels and offering valuable insights into plant adaptations in saline environments.

The utilization of Partial Least Squares (PLS) analysis validated the statistical impact of soil parameters on carbohydrate accumulation^20^, This shows the significant correlations between soil characteristics including Electrical Conductivity (EC), pH, and chloride (Cl^−^) content and plant species sugar content, suggesting plant-microbe interactions. *Sueda nudiflora*, *Aeluropus lagopoides*, and *Avicennia marina* had higher EC and Cl^−^ levels, indicating salinity stress, and increased sugar accumulation, likely as an osmotic adjustment mechanism to counteract saline pressure. Soil microorganisms help plants absorb nutrients, produce growth-promoting substances, and break down organic matter, that results into modifying soil properties like pH, salinity, and nutrient availability. The PLS correlations suggest that rhizosphere microbial activity may affect soil characteristics and plant sugar metabolism. Symbiotic relationships occur when plant roots release sugars and other organic compounds that feed soil microbes, which improve plant nutrient acquisition and stress resilience. Some soil microbes produce hormones or other signalling molecules that increase plant metabolism and photosynthetic activity, producing sugar. The PLS analysis indicates that soil characteristics directly affect sugar content and that plant-microbe interactions are crucial to plant adaptation. Microbial activity modulates soil properties, enhancing these halophytes and mangroves’ carbohydrate accumulation strategies and supporting their survival and growth in saline environments.

K-means cluster analysis was applied to FT-IR data to ascertain the similarity index of cluster configuration^46^. Overall, this investigation indicates that the clustering algorithm effectively segregated FT-IR data into two distinct clusters, each characterized by unique spectral properties at their respective cluster centers. Further analysis may involve scrutinizing the spectral attributes linked to each cluster and exploring any significant patterns or discrepancies.

Recent research has established a link between elevated osmolytic sugar levels in halophytes and mangroves, resulting in reduced ROS generation and enhanced salt stress tolerance. Future studies should delve into the physiological and molecular mechanisms governing sugar metabolism in these plants^4^ and explore the specific functions of different sugars in stress adaptation.

## Conclusion

This study elucidates the intricate relationship between the dynamics of complex carbohydrates and the tolerance of coastal flora to abiotic stress. The halophytes *S. nudiflora* and *A. lagopoides*, as well as the mangrove *A. marina*, exhibited distinct patterns of sugar accumulation. Halophytes exhibited pronounced seasonal variations in sugar levels, particularly during the monsoon period. These modifications could be adjustments made for the purpose of plant-microbe interactions in the root area. Monsoons enhance the availability of freshwater, nutrients, and microbial activity. The current surge in sugar production is likely to promote the growth of advantageous microbial communities. These microorganisms can enhance the absorption of nutrients by plants, particularly in saline environments, and alleviate stress by producing compatible solutes or enhancing resistance to pathogens. Nevertheless, the consistent sugar levels of A. marina throughout the year indicate the presence of alternative mechanisms for tolerating stress. These mechanisms may be connected to pneumatophores, which are specialised aerial roots that facilitate the absorption of oxygen in soils with low oxygen levels. Pneumatophores are a typical characteristic of mangrove ecosystems.

The adaptability of coastal plants is demonstrated by the varying dynamics of carbohydrates. Additional investigation utilizing stable isotope probing can elucidate the functions of various sugars in these interactions. This technique enables researchers to trace isotopically labeled carbon substrates across the food chain in order to identify the specific microbes that utilize plant sugars. Metagenomic analysis of rhizosphere microbial communities can provide insights into the composition and activities of these communities in different seasons and with different plant species. In order to comprehend the ability of organisms to withstand stress in saline environments, it is necessary to grasp the impact of carbohydrate distribution on the structure and function of these communities.

This information can aid in the development of sustainable management strategies that enhance the health and resilience of these crucial ecosystems. Optimal soil conditions that promote advantageous microbial communities can enhance plant resilience to stress and minimize the need for fertilizers. Understanding the intricate connection between plants and their microbial counterparts has the potential to pave the way for groundbreaking biotechnologies that enhance crop productivity in challenging environments. Strategic manipulation of plant-microbe interactions has the potential to enable the development of crops that are able to tolerate high levels of salt and resist various types of stress.

## Electronic supplementary material

Below is the link to the electronic supplementary material.

Supplementary Material 1

## Data Availability

The data that support the findings of this study are not openly available due to reasons of sensitivity and are available from the corresponding author upon reasonable request.

## References

[1] Banerjee, K., Sappal, S. M., Ramachandran, P. & Ramesh, R. Salt Marsh: Ecologically important, yet least studied blue carbon ecosystems in India. *J. Clim. Change*. **3** (2), 59–72 (2017). 10.3233/JCC-170014

[2] Kumari, A. & Rathore, M. S. Roles of mangroves in combating the climate change. In Mangroves: Ecology, *Biodiversity and Management* (pp. 225–255). *Springer*. (2021).

[3] Vyas, S., Agoramoorthy, G., Gadhvi, K., Gamit, S. & Dangar, K. Correlation of elemental hyperaccumulation among the succulent and non-succulent halophytes of Gujarat, India. *Sci. Rep. ***13** (1), 16361 (2023). 37773347 10.1038/s41598-023-42980-8 PMC10541406

[4] Lokhande, V. H. & Suprasanna, P. Prospects of halophytes in understanding and managing abiotic stress tolerance. In Environmental Adaptations and Stress Tolerance of Plants in the Era of Climate Change (pp. 29–56). *Springer*. (2012).

[5] Wungrampha, S., Rawat, N., Singla-Pareek, S. L. & Pareek, A. Survival strategies in halophytes: Adaptation and regulation. In Handbook of halophytes: From molecules to ecosystems towards biosaline agriculture (1591–1612). Cham: Springer International Publishing (2020).

[6] Naskar, K. & Mandal, R. Ecology and biodiversity of Indian mangroves (Vol. 1). Daya. (1999).

[7] Mehta, D. & Vyas, S. Comparative bio-accumulation of osmoprotectants in saline stress tolerating plants: A review. *Plant. Stress*, **100177**. (2023).

[8] Chavda, N. H., Chabhadiya, V. K., Pithawala, E. A. & Pandya, J. B. Plant diversity of ghogha coastal area, dist. Bhavnagar. *Int. J. Sci. Res. Archive*. **9** (1), 566–570 (2023). 10.30574/ijsra.2023.9.1.0474

[9] Mohammed, S. H. E. R., Kasera, P. K., Chawan, D. D. & Sen, D. N. Osmotic potential in the leaf sap of halophytes in Indian arid zone. *J. Indian Bot. Soc. ***77**, 179–184 (1998).

[10] Hendrix, D. L. & Pierce, W. S. Osmoregulation and membrane-mediated responses to altered water potential in plant cells. *Cryobiology*. **20** (4), 466–486 (1983). 6352178 10.1016/0011-2240(83)90036-6

[11] Rozentsvet, O. A. et al. Structural and functional organization of the photosynthetic apparatus in halophytes with different strategies of salt tolerance. *Photosynthetica*. **54** (3), 405–413 (2016). 10.1007/s11099-015-0182-6

[12] Parida, A. K., Das, A. B. & Mohanty, P. Investigations on the antioxidative defence responses to NaCl stress in a mangrove, Bruguiera parviflora: Differential regulations of isoforms of some antioxidative enzymes. *Plant. Growth Regul. ***42**, 213–226 (2004). 10.1023/B:GROW.0000026508.63288.39 15202709

[13] Poljakoff-Mayber, A. & Lerner, H. R. Plants in saline environments. Handbook of plant and crop stress, 2, 125–154 (1999).

[14] Ponpiboon, T. & Vichkovitten, T. Seasonal variation on photosynthetic pigment, soluble sugars and starch contents of mangrove (Avicennia Alba Bl). *GMSARN Int. J. ***13**, 112–118 (2019).

[15] Mohammadkhani, N. & Heidari, R. Drought-induced accumulation of soluble sugars and proline in two maize varieties. *World Appl. Sci. J. ***3** (3), 448–453 (2008).

[16] Gous, P. W., Gilbert, R. G. & Fox, G. P. Drought-proofing Barley (Hordeum vulgare) and its impact on grain quality: A review. *J. Inst. Brew. ***121** (1), 19–27 (2015). 10.1002/jib.187

[17] Morsy, M. R., Jouve, L., Hausman, J. F., Hoffmann, L. & Stewart, J. M. Alteration of oxidative and carbohydrate metabolism under abiotic stress in two rice (Oryza sativa L.) genotypes contrasting in chilling tolerance. *J. Plant Physiol. ***164** (2), 157–167 (2007). 16500726 10.1016/j.jplph.2005.12.004

[18] Saddhe, A. A., Manuka, R. & Penna, S. Plant sugars: Homeostasis and transport under abiotic stress in plants. *Physiol. Plant. ***171** (4), 739–755 (2021). 33215734 10.1111/ppl.13283

[19] Smith, J. L. & Doran, J. W. Measurement and use of pH and electrical conductivity for soil quality analysis. *Methods Assess. Soil Qual. ***49**, 169–185 (1997).

[20] Kawamura, K. et al. Vis-NIR spectroscopy and PLS regression with waveband selection for estimating the total C and N of paddy soils in Madagascar. *Remote Sens. ***9** (10), 1081 (2017). 10.3390/rs9101081

[21] Subbarao, G. V., Nam, N. H., Chauhan, Y. S. & Johansen, C. Osmotic adjustment, water relations and carbohydrate remobilization in pigeonpea under water deficits. *J. Plant Physiol. ***157** (6), 651–659 (2000). 10.1016/S0176-1617(00)80008-5

[22] Akao, K. I., Okubo, Y., Asakawa, N., Inoue, Y. & Sakurai, M. Infrared spectroscopic study on the properties of the anhydrous form II of trehalose. Implications for the functional mechanism of trehalose as a stabilizer. *Carbohydr. Res. ***334** (3), 233–241 (2001). 11513830 10.1016/S0008-6215(01)00182-3

[23] Seedevi, P. et al. Isolation and chemical characteristics of rhamnose enriched polysaccharide from Grateloupia lithophila. *Carbohydrate polymers*, 195, 486–494 Joshi, A. J. *Monograph on Indian halophytes*. Ocean & Atmospheric Science and Technology Cell. Dept. of Life Science, Bhavnagar University, India, 140 (2011). (2018). 10.1016/j.carbpol.2018.05.002 29805003

[24] Shah, G. L. *Flora of Gujarat State (Vols. 1)* (University Press, Sardar Patel University, 1978).

[25] Modi, D. S., Bhandari, S. N. & Zala, L. B. Land feature extraction-identification and discrimination using geospatial techniques. *Kalpa Publications Civil Eng. ***1**, 248–258 (2017). 10.29007/ckgd

[26] Pandya, J., Kheni, M. P., Jani, R. B. & Mehta, S. K. *Sea water & coastal soil analysis from selected coastal areas of Bhavnagar district, Gujarat, India* (International Association of Biologists and Computational Digest, 2022).

[27] World Health Organization. *WHO guidelines on good agricultural and collection practices [GACP] for medicinal plants* (Philippines. World Health Organization, 2003).

[28] Joshi, A. J. *Monograph on Indian halophytes. Ocean & Atmospheric Science and Technology Cell. Department of Life Science*140 (Bhavnagar University, 2011).

[29] Vadera, H. R., Pandya, J. B. & Mehta, S. K. Quantitative analysis of source-sink relationship in leaves and fruit of Cucumis melo L. *Int. J. Researches Biosci. Agric. Technol. ***17**, 356–365 (2021).

[30] Hansen, J. & Møller, I. B. Percolation of starch and soluble carbohydrates from plant tissue for quantitative determination with anthrone. *Anal. Biochem. ***68** (1), 87–94 (1975). 1190454 10.1016/0003-2697(75)90682-X

[31] Clegg, K. M. The application of the anthrone reagent to the estimation of starch in cereals. *J. Sci. Food. Agric. ***7** (1), 40–44 (1956). 10.1002/jsfa.2740070108

[32] Hashimoto, A., Nakanishi, K., Motonaga, Y. & Kameoka, T. Sugar metabolic analysis of suspensions of plant cells using an FT-IR/ATR method. *Biotechnol. Prog. ***17** (3), 560–564 (2001). 11386879 10.1021/bp010013w

[33] Yancey, P. H., Clark, M. E., Hand, S. C., Bowlus, R. D. & Somero, G. N. Living with water stress: Evolution of osmolyte systems. *Science ***217** (4566), 1214–1222 (1982). 7112124 10.1126/science.7112124

[34] Mi, F. L. et al. Synthesis and characterization of a novel glycoconjugated macromolecule. *Polymer ***47** (12), 4348–4358 (2006). 10.1016/j.polymer.2006.04.005

[35] Hincha, D. K., Hellwege, E. M., Heyer, A. G. & Crowe, J. H. Plant fructans stabilize phosphatidylcholine liposomes during freeze-drying. *Eur. J. Biochem. ***267** (2), 535–540 (2000). 10632723 10.1046/j.1432-1327.2000.01028.x

[36] Lunn, J. E., Delorge, I., Figueroa, C. M., Van Dijck, P. & Stitt, M. Trehalose metabolism in plants. *Plant J. ***79** (4), 544–567 (2014). 24645920 10.1111/tpj.12509

[37] Paul, M. J., Primavesi, L. F., Jhurreea, D. & Zhang, Y. Trehalose metabolism and signaling. *Annu. Rev. Plant Biol. ***59**, 417 (2008). 18257709 10.1146/annurev.arplant.59.032607.092945

[38] Konstantinova, T., Parvanova, D., Atanassov, A. & Djilianov, D. Freezing tolerant tobacco, transformed to accumulate osmoprotectants. *Plant Sci. ***163** (1), 157–164 (2002). 10.1016/S0168-9452(02)00090-0

[39] Vijn, I. & Smeekens, S. Fructan: More than a reserve carbohydrate? *Plant Physiol. ***120** (2), 351–360 (1999). 10364386 10.1104/pp.120.2.351 PMC1539216

[40] Shen, B., Jensen, R. G. & Bohnert, H. J. Mannitol protects against oxidation by hydroxyl radicals. *Plant Physiol. ***115** (2), 527–532 (1997). 12223821 10.1104/pp.115.2.527 PMC158511

[41] Jiang, N., Dillon, F. M., Silva, A., Gomez-Cano, L. & Grotewold, E. Rhamnose in plants-from biosynthesis to diverse functions. *Plant Sci. ***302**, 110687 (2021). 33288005 10.1016/j.plantsci.2020.110687

[42] ElSayed, A. I., Rafudeen, M. S. & Golldack, D. Physiological aspects of raffinose family oligosaccharides in plants: Protection against abiotic stress. *Plant Biol. ***16** (1), 1–8 (2014). 23937337 10.1111/plb.12053

[43] O’Neill, R. V. *A hierarchical concept of ecosystems No. 23* (Princeton University Press, 1986).

[44] Matros, A. et al. Genome-wide association study reveals the genetic complexity of fructan accumulation patterns in barley grain. *J. Exp. Bot. ***72** (7), 2383–2402 (2021). 33421064 10.1093/jxb/erab002

[45] Granato, D., Santos, J. S., Escher, G. B., Ferreira, B. L. & Maggio, R. M. Use of principal component analysis (PCA) and hierarchical cluster analysis (HCA) for multivariate association between bioactive compounds and functional properties in foods: A critical perspective. *Trends Food Sci. Technol. ***72**, 83–90 (2018). 10.1016/j.tifs.2017.12.006

[46] Wander, L. et al. Exploratory analysis of hyperspectral FTIR data obtained from environmental microplastics samples. *Anal. Methods ***12** (6), 781–791 (2020). 10.1039/C9AY02483B

[47] Li, M. H. et al. Mobile carbohydrates in himalayan treeline trees I. Evidence for carbon gain limitation but not for growth limitation. *Tree Physiol. ***28** (8), 1287–1296 (2008). 18519260 10.1093/treephys/28.8.1287

