# Supplementary material for: Carbohydrate accumulation patterns in mangrove and halophytic plant species under seasonal variation
Source: Sci Rep. 2024 Sep 14;14:21512. doi: 10.1038/s41598-024-72627-1 (PMC11401893; doi:10.1038/s41598-024-72627-1)
Supplement: Supplementary file 1 — Supplementary Material 1 [file 41598_2024_72627_MOESM1_ESM.docx]

**Supplementary Figure 1A: FT-IR of carbohydrate molecules in *A.lagopoides***

**Supplementary Figure 1B: FT-IR of carbohydrate molecules in *S.nudiflora***

**Supplementary Figure 1C: FT-IR of carbohydrate molecules in *A.marina***
